# EZH2-dependent epigenetic reprogramming in the central nucleus of amygdala regulates adult anxiety in both sexes after adolescent alcohol exposure

**DOI:** 10.1038/s41398-024-02906-y

**Published:** 2024-04-26

**Authors:** John Peyton Bohnsack, Huaibo Zhang, Subhash C. Pandey

**Affiliations:** 1 Center for Alcohol Research in Epigenetics, Department of Psychiatry, University of Illinois Chicago, Chicago, IL 60612 USA; 2 Jesse Brown Veterans Affairs Medical Center, Chicago, IL 60612 USA; 3 Department of Anatomy and Cell Biology, University of Illinois Chicago, Chicago, IL 60612 USA

**Keywords:** Epigenetics and behaviour, Addiction

## Abstract

Alcohol use and anxiety disorders occur in both males and females, but despite sharing similar presentation and classical symptoms, the prevalence of alcohol use disorder (AUD) is lower in females. While anxiety is a symptom and comorbidity shared by both sexes, the common underlying mechanism that leads to AUD and the subsequent development of anxiety is still understudied. Using a rodent model of adolescent intermittent ethanol (AIE) exposure in both sexes, we investigated the epigenetic mechanism mediated by enhancer of zeste 2 (EZH2), a histone methyltransferase, in regulating both the expression of activity-regulated cytoskeleton-associated protein (Arc) and an anxiety-like phenotype in adulthood. Here, we report that EZH2 protein levels were significantly higher in PKC-δ positive GABAergic neurons in the central nucleus of amygdala (CeA) of adult male and female rats after AIE. Reducing protein and mRNA levels of EZH2 using siRNA infusion in the CeA prevented AIE-induced anxiety-like behavior, increased H3K27me3, decreased H3K27ac at the *Arc* synaptic activity response element (SARE) site, and restored deficits in *Arc* mRNA and protein expression in both male and female adult rats. Our data indicate that an EZH2-mediated epigenetic mechanism in the CeA plays an important role in regulating anxiety-like behavior and *Arc* expression after AIE in both male and female rats in adulthood. This study suggests that EZH2 may serve as a tractable drug target for the treatment of adult psychopathology after adolescent alcohol exposure.

## Introduction

Alcohol use disorder (AUD) is a severe and debilitating psychiatric disease that is responsible for 95,158 deaths in the United States each year from 2011–2015 [1]. A major risk factor for developing an AUD is the consumption of alcohol during adolescence [2, 3]. Several studies have demonstrated that adolescent alcohol exposure induces persistent behavioral and molecular changes that lead to adult AUD, in addition to other comorbid disorders such as anxiety [4–6]. Men are more likely to have an AUD (16.7%) than women (9.0%) [7, 8]. Conversely, anxiety disorders are more prevalent in women (33.3%) than in men (22.0%) with no difference in age of first occurrence [9]. Over the past 10 years, the gap between the prevalence of male and female AUD diagnoses has decreased, and the rate of AUDs in women is growing at a higher rate (84%) than in men (35%) [7, 8]. A large body of literature suggests that for both humans and rodents, females are more susceptible to developing AUD due to early life stress, which is predictive of a greater potential to continue to have AUD later in life as well as a higher risk of relapse [10]. Individuals diagnosed with AUD are 20-40% more likely to also have a comorbid anxiety disorder [11]. Human and rodent studies have demonstrated that alcohol withdrawal causes anxiety and contributes to the likelihood of relapse [4, 11–14].

The central nucleus of the amygdala (CeA) is a critical brain region that undergoes changes during adolescence [15] and is heavily implicated in AUD [4, 16, 17]. Adolescent consumption of alcohol disrupts normal amygdala function, which can increase the risk of developing an AUD and other psychopathology [6, 18, 19]. The CeA is involved in regulating anxiety behaviors and contains protein kinase C-delta (PKC-δ) positive GABAergic neurons that are inhibitory in nature [17, 20–22]. Activity-regulated cytoskeleton-associated protein (Arc) is a critical regulator of synaptic plasticity and neurotransmission [23]. *Arc* is an immediate early gene that responds to synaptic activity via an enhancer region located ~7 kb upstream from the transcription start site, which is known as the synaptic activity response element (SARE) [24]. *Arc* is down-regulated in both human AUD postmortem amygdala [25] and rodent models of alcohol dependence [26, 27]. Knockdown of *Arc* in the CeA increases alcohol consumption and anxiety-like behaviors in adult male rats [12, 26]. Adult rats exposed to adolescent intermittent ethanol (AIE) show decreased *Arc* mRNA expression in the amygdala and increased anxiety-like behaviors in adulthood [26, 27]. These studies suggest that the CeA plays a critical role in regulating the effects of adolescent alcohol exposure, including comorbid anxiety and anxiety that is caused by alcohol dependence.

Epigenetic mechanisms play an important role in the regulation of gene expression related to synaptic plasticity in AUD (4,6,25). Enhancer of zeste homolog 2 (EZH2) is the catalytic unit of the polycomb repressive complex 2 (PRC2), which deposits H3K27me3 and results in transcriptional repression [28]. EZH2 also plays an important role in neural development by guiding cell-fate decisions via temporal gene repression [29, 30]. In adulthood, EZH2 is essential in neurogenesis, memory, and anxiety [31, 32]. Previous studies have found increased repressive H3K27me3 to be associated with the *Arc* SARE after AIE, and that increasing H3K27me3 by knocking down KDM6B in CeA induces anxiety-like behavior in adult ethanol-naïve rats [26, 33]. Another study conducted using human postmortem amygdala of individuals with AUD found that EZH2 mediates substantial repressive epigenetic remodeling and increases H3K27me3 at the *ARC* SARE, which corresponds with decreased *ARC* expression in individuals who began drinking during adolescence (early onset) [25]. This suggests that H3K27me3 in the amygdala might be associated with the long-lasting effects of adolescent alcohol exposure in both humans and rodents. Recently, epigenomic editing of the *Arc* SARE site using dCas9-P300 in the CeA ameliorated anxiety and alcohol drinking behaviors and normalized the deficit in *Arc* expression in male rats after AIE in adulthood [33]. However, there is not enough literature that evaluates the effect of adolescent alcohol exposure on adult anxiety-related behaviors in both male and female preclinical models or whether EZH2 regulates this phenotype and *Arc* expression via epigenetic modifications of the *Arc* SARE site in both sexes. Here, to determine if there is a sex-specific difference in epigenetic modifications and expression of *Arc* gene, we evaluated the role of EZH2 in the CeA in driving anxiety-like behavior in adulthood after AIE.

## Materials and methods

### Animals and adolescent intermittent ethanol exposure

Sprague Dawley dams (Harlan) with pups were shipped to the University of Illinois Chicago (arrival postnatal day, PND 17). Pups were weaned on PND 21 then separated by sex, and group-housed (2-3/cage) under a 12:12 light dark/cycle with *ab libitum* access to food and water. All animal experimental protocols adhered to the NIH Guidelines for the Care and Use of Laboratory Animals and were approved by University of Illinois Chicago Institutional Animal Care and Use Committee.

Adolescent intermittent ethanol (AIE) or saline (AIS) exposure was performed as we have previously described [26, 27]. Starting on PND 28, rats were randomized to receive either 2 g/kg EtOH (20% w/v in 0.9% NaCl) or volume matched vehicle (0.9% NaCl) every 2 days until PND 41, after which they were allowed to mature until adulthood.

### Stereotaxic surgery and EZH2 siRNA infusion

Stereotaxic surgery was performed as previously described [26, 33]. AIE and AIS adult rats were anesthetized with isoflurane (3%) and bilaterally cannulated into the CeA (from bregma, posterior -2.5, medial-lateral ±4.2, ventral -5.1), then allowed minimum one-week recovery. Rats were housed singly after surgery and monitored daily. EZH2 siRNA (Qiagen, #SI01727747, Rn_LOC312299_1; Sense strand 5’CCUCAAUGUUUCCAGAUAATT-3’, Antisense strand 5’UUAUCUGGAAACAUUGAGGAA-3’) or negative control siRNA (Qiagen, # 1027310) were dissolved in sterile H_2_O and mixed with i-Fect solution (Neuromics, NI35750) at a 1:7 ratio to provide a final concentration of 2 µg/uL. No off targets were identified after checking of siRNA sequence for homology to all other sequences of the genome (Qiagen). 0.5 µL (1 µg) was slowly infused once into each side of the CeA using probe that targets 3 mm beyond guided cannula. 24 hours later, animals were subjected to behavioral testing and then were immediately given anesthesia (3% isoflurane) and sacrificed to collect brains. The amygdala (predominantly the CeA, but also some of the surrounding MeA and BLA) was micro dissected and then flash frozen on dry ice and stored at -80 °C until biochemical analysis. The dose of siRNA and the time course were based on our earlier publications [26, 34]. Another cohort of animals was anesthetized with 3% isoflurane then perfused with 4% paraformaldehyde in 0.1 M phosphate buffer (pH 7.4). After fixation, brains were soaked in a sucrose gradient (10%, 20%, 30%) then flash frozen in 2-methylbutane (−20 °C to −30 °C), after which they were stored at −80 °C and used for immunohistochemistry as described below.

### Elevated plus-maze (EPM) exploration test

The elevated plus maze test was performed as previously described [26, 27, 35]. Animals were moved to the experimental room for 10 minutes to acclimate to the experimental environment, and then they were placed in the elevated plus maze and allowed to explore for 5 minutes. Number of entries and time spent in both the open and closed arms were recorded. Percentage of time spent on open arms was calculated from total time spent in open and closed arms of the EPM.

### Light/dark box (LDB) exploration test

The light/dark box exploratory test was performed as previously described [26, 27, 35]. Rats were moved to the experimental room for 10 minutes to acclimate to the experimental environment. After this they were placed in the light/dark box and allowed to explore for 5 minutes. The amount of time spent in light and dark boxes was recorded along with total ambulation using an infrared tracking system (San Diego Instruments). Percentage of time spent in the light box was calculated from the total time spent in both light and dark boxes.

### qPCR procedure to measure mRNA expression

qPCR was performed as previously described [25, 26]. RNA was extracted by homogenizing amygdala tissue in Trizol and then purified using Micro Direct-zol Purification kit following manufacturer’s instructions (Zymo). RNA was reverse transcribed to cDNA using MultiScribe Reverse Transcriptase (ThermoFisher Scientific) following manufacturer’s instructions. qPCR reactions were run on a CFX-Connect qPCR system using Powerup SYBR (Thermo Scientific). Changes in expression were determined using the ∆∆Ct method and normalized to mean of Ct values of the *Hprt1* housekeeping gene. Data are presented as average fold change relative to controls. Primer sequences have been previously published [26, 33].

### Chromatin immunoprecipitation (ChIP)-qPCR

Chromatin immunoprecipitations were performed as previously described [25, 33, 36]. Amygdala tissue was homogenized in PBS then cross-linked with 1% formaldehyde for 10 minutes at room temperature, quenched with 1 M glycine in 750 mM Tris HCl pH 8.0, then centrifuged at 1,600×g for 10 minutes at 4 °C and washed once with ice-cold PBS. Tissues were lysed and chromatin fraction was prepared according to procedure previously published by us [33, 36]. Antibodies (EZH2, 2 µg, Active Motif #39875; H3K27me3, 2 µg, Active Motif #39155; H3K27ac, 2 µg, Active Motif #39133) were added to chromatin and then were rotated overnight at 4 °C. 30 µL of Dynabeads A were added and rotated for 1 hr at 4 °C. Samples were washed 5 times with ChIP washing buffer then purified using 10% w/v Chelex 100 (Biorad) in sterile H_2_O by boiling for 95 °C for 10 minutes followed by centrifugation. Input samples were purified by centrifugation followed by washing once with 75% ethanol, then boiled for 10 minutes at 95 °C in 10% w/v Chelex, and purified DNA was then used for qPCR. The data were analyzed using the ∆∆Ct method, normalized to input, and are expressed as fold change in protein occupancy. Primers for the *Arc* SARE site have been previously published by us [26, 33].

### Measurement of protein levels using immunohistochemistry (IHC)

Immunogold labeling was performed as previously described [26, 27, 35]. Brains were sliced to 20 µm sections on a cryostat and washed 3 times with PBS, and then they were blocked with 10% normal goat serum, containing 0.25% Triton X-100, in PBS for 30 min at RT. Sections were then incubated with 1% BSA (prepared in PBS containing 0.25% Triton X-100) for 30 min at room temperature. Later, Bregma matched sections were incubated in primary antibody [Arc (1:200 dilution), Synaptic Systems #156003; EZH2 (1:200 dilution), Proteintech Group #21800-1-AP] for at least 18 h at room temperature. After two washes for 10 min each with PBS and two washes for 10 min each with 1% BSA in PBS, sections were incubated with gold particle (1.4 nm) conjugated anti-rabbit or anti-mouse secondary antibody (Nanoprobes, 1:200 dilution in 1% BSA in PBS) for 1 h at room temperature. Further, sections were rinsed three times in 1% BSA in PBS followed by extensive rinsing in double-distilled water. The gold immunolabeling was developed using silver enhancement solution (Ted Pella) for 15–20 min, and then sections were washed three times using tap water. Sections were then mounted on slides and quantification was performed using the Image Analysis System (Loats Associates) connected to a light microscope. The threshold was set so that the area without staining should give zero counts. Immunogold particles from three adjacent brain sections (nine total object fields) of each rat were counted at high magnification (100×), and then values were averaged for each rat. The results were presented as the number of immunogold particles/100 μm^2^ area.

### Double Immunofluorescence Staining

Brains were perfused and sliced to 20 µm sections on a cryostat as described above. The coronal brain sections were washed twice with 0.01 M PBS for 10 minutes each. Sections were incubated with 10% normal donkey serum (NDS) diluted in PBST for 30 minutes and then blocked with 1% BSA prepared in PBST for 30 minutes at room temperature. Sections were incubated with primary antibody for PKC-δ (anti-mouse, BD Biosciences, catalog number 610398) and EZH2 (anti-rabbit, Proteintech, catalog number 21800-1-AP) with 1:200 dilution in 3% NDS in PBST overnight at 4 °C. Following incubation with primary antibodies, these sections were washed 3 × 10-minutes with 0.01 M PBS. Sections were then incubated in the dark with conjugated anti-mouse antibody (Jackson ImmunoResearch, Alexa Fluor 594 conjugate red color, catalog number 715-585-150) and anti-rabbit (Alexa Fluor 488 green color, catalog number 711-545-152) with 1:500 dilution each in 0.01 M PBST for 2 hours at room temperature. Sections were washed, mounted on slides, air-dried, and cover-slipped using Fluoromount-G (Invitrogen, catalog number 00-4958-02). Slides were kept at 4 °C. Three images per animal were taken using a confocal microscope (LSM 710; Zeiss, Thornwood, NY, USA) at 40x magnification for each brain region (CeA, MeA, and BLA). Fluorescence intensities for overall EZH2 positive cells as well as co-expressing EZH2 in PKC-δ-positive or negative cells were quantified using the integrated density function in Fiji ImageJ (NIH). Integrated density values were then averaged for each animal and data were calculated per 100 µm^2^ and then expressed as percent of control.

### Statistics

Sample size was determined based on our previous studies in AIE model (26,27,33). Animals were assigned to various groups randomly. Statistical analyses were performed using SigmaStat 3.5 (Systat Software). Two-way ANOVA was used for comparisons between four groups followed by Tukey’s post hoc test. Comparisons between two groups were performed using Student’s t test. The outliers were determined using Grubbs’ Test.

## Results

### EZH2 is upregulated in the CeA and MeA after AIE in adult rats

**Fig. 1:**
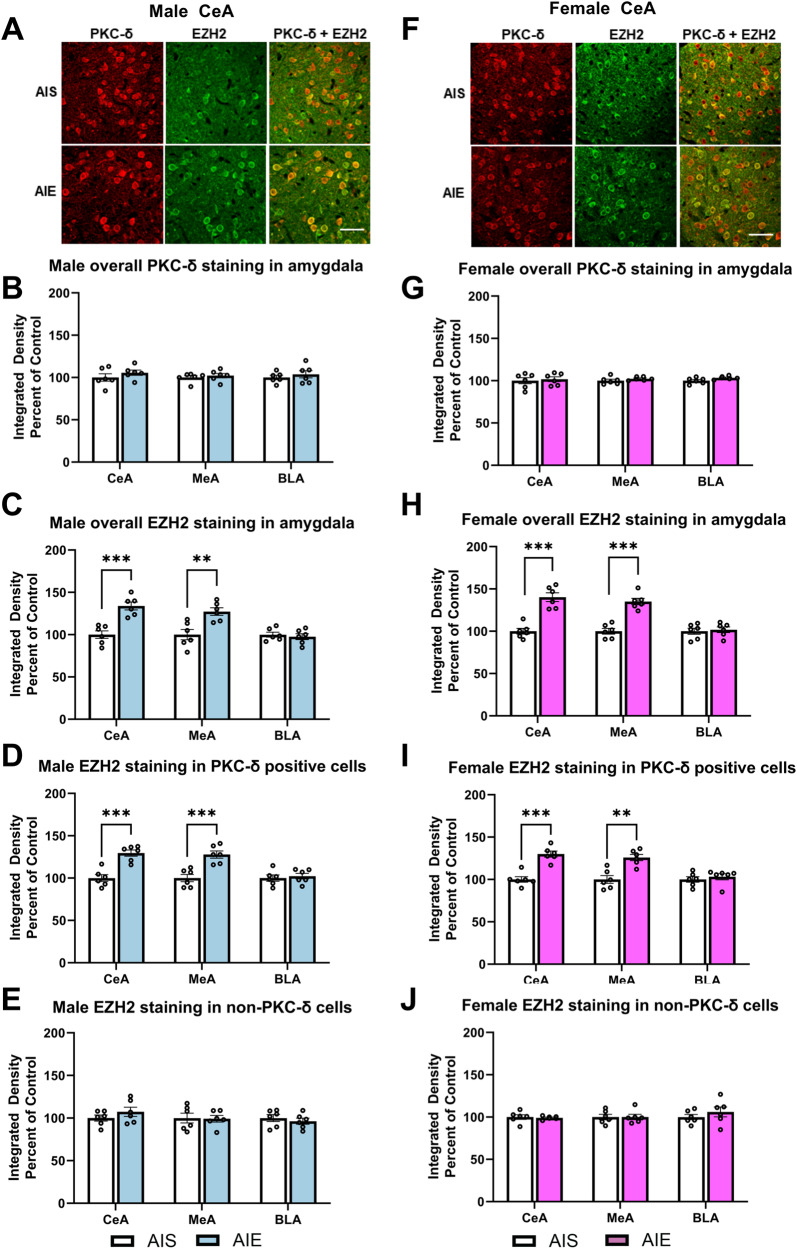
Adolescent intermittent ethanol (AIE) exposure increases EZH2 in PKC-δ positive cells in the central (CeA) and medial (MeA) nucleus of amygdala. **A** Representative photomicrograph of fluorescent immunostaining images of EZH2 and PKC-δ in the CeA of AIE and AIS adult male rats (Scale bar = 50 µm). **B** AIE has no effects on PKC-δ protein levels in the CeA, MeA, and basolateral amygdala (BLA) when compared with AIS adult male rats. **C** AIE increases overall EZH2 protein levels in the CeA and MeA, but not the BLA as compared with AIS adult male rats. **D** AIE increases EZH2 protein levels in PKC-δ positive cells in the CeA and MeA, but not the BLA as compared with AIS adult male rats. **E** AIE has no effects on EZH2 protein levels in non- PKC-δ cells the CeA, MeA, and BLA as compared with AIS adult male rats. **F** Representative photomicrograph of fluorescent immunostaining images in the CeA of AIE and AIS adult female rats (Scale bar = 50 µm). **G** AIE has no effects on PKC-δ protein levels in the CeA, MeA, and BLA as compared with AIS adult female rats. **H** Similar to male rats, AIE increases overall EZH2 expression in the CeA and MeA, but not the BLA as compared with AIS adult female rats. **I** AIE increases EZH2 expression in PKC-δ positive cells of the CeA and MeA, but not the BLA as compared with AIS adult female rats. **J** AIE has no effects on EZH2 protein levels in non-PKC-δ cells in the CeA, MeA, and BLA as compared with AIS adult female rats. Statistical significance was determined with Student’s *t*-test (**p < 0.01, ***p < 0.001). Values are mean ± SEM. *n* = 6 per group. Individual value for each rat is represented by a circle dot on the bar diagram. AIS Adolescent intermittent saline, PKC-δ Protein kinase C delta.

We wanted to assess if there are differences in EZH2 expression within the adult amygdala after adolescent intermittent ethanol (AIE) in male and female rats. Overall, EZH2 expression is increased in the CeA (Male, *t* = 5.25, df = 10, *p* < 0.001; Female, *t* = 6.52, df = 10, *p* < 0.001) and MeA (Male, *t* = 3.65, df=10, *p* < 0.01; Female, *t* = 6.98,df = 10, *p* < 0.001) but not in BLA after AIE, whereas immunofluorescent staining of PKC-δ was not altered in the CeA, MeA, and BLA after AIE in male (Fig. 1A–C) and female rats (Fig. 1F–H). Our results also found that for male rats, there is an increase in EZH2 expression on PKC-δ positive GABAergic neurons in both the CeA (*t* = 5.38, df = 10, *p* < 0.001) and MeA (*t* = 4.63, df = 10, *p* < 0.001), but not in the BLA (Fig. 1D). In female rats, a similar increase in EZH2 on PKC-δ positive GABAergic neurons was observed in both the CeA (*t* = 6.22, df = 10, *p* < 0.001) and MeA (*t* = 4.44, df = 10, *p* < 0.01), but not in the BLA (Fig. 1I). Interestingly, EZH2 expression on non-PKC-δ cells in the CeA, MeA, and BLA (Fig. 1E, J) of adult male and female rats after AIE was not altered. These results suggest that EZH2 protein levels are increased in PKC-δ positive GABAergic neurons in the CeA and MeA of adult male and female rats after AIE exposure.

### EZH2 knockdown in the CeA prevents anxiety-like behavior after AIE in adulthood in both sexes

**Fig. 2:**
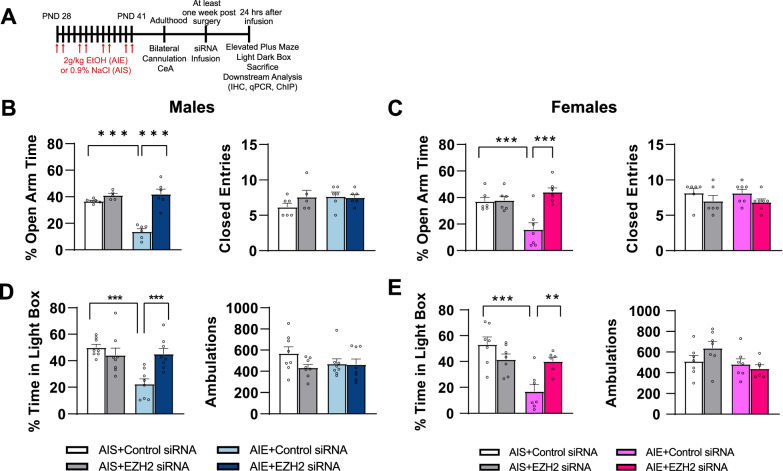
EZH2 knockdown in the CeA prevents AIE-induced anxiety-like behaviors in adulthood. **A** Experimental schematic outlining adolescent intermittent ethanol (AIE) and saline (AIS) procedures, progression into adulthood, surgery schedule, and molecular and behavioral analysis. **B** AIE induces adulthood anxiety-like behavior in male rats that is prevented by knockdown of EZH2 in the central nucleus of amygdala (CeA) in the elevated plus maze (EPM) test (*n* = 5–6/group). **C** AIE induces adulthood anxiety-like behavior in female rats that is prevented by the knockdown of EZH2 in the CeA, in the EPM test (*n* = 6–7/group). **D** AIE induces adulthood anxiety-like behavior in male rats during the light/dark box (LDB) test that can be prevented by EZH2 knockdown in the CeA (*n* = 8/group). **E** AIE induces adulthood anxiety-like behavior in female rats in the LDB test that can be prevented by EZH2 knockdown in the CeA (*n* = 6–7/group). Values are mean ± SEM. Statistical significance was determined with two-way ANOVA followed by Tukey’s post hoc test (**p < 0.01, ***p < 0.001). The individual value for each rat is represented by a circle dot on the bar diagram. AIS Adolescent intermittent saline, AIE Adolescent intermittent ethanol, PND Postnatal day.

We tested whether AIE would produce anxiety-like behaviors in female rats similar to those previously observed in male rats [26, 27, 33] as well as whether EZH2 knockdown in the CeA would prevent AIE-induced anxiety-like behaviors in adulthood (Fig. 2A). In both male (Two-way ANOVA, group x siRNA infusion interaction; F_1,19_ = 24.52, *p* < 0.001) and female (Two-way ANOVA, group x siRNA infusion interaction; F_1,22_ = 12.8, *p* < 0.01) rats, we observed AIE produced anxiety-like behavior (reduction in % time spent in open arm) in the EPM in adulthood (male, Fig. 2B; female, Fig. 2C). EZH2 siRNA infusion into CeA attenuated AIE-induced anxiety-like behaviors in the EPM test in both male and female rats (Fig. 2B, C). EZH2 siRNA infusion had no effect on anxiety-like behaviors measured with EPM in male and female rats exposed to saline (AIS) (Fig. 2B, C). We used a second measure of anxiety, the LDB exploration test, to validate EPM findings in a separate cohort of rats. We found that AIE produced anxiety-like behavior (reduction in % time spent in light box) in both male (Two-way ANOVA, group x siRNA infusion interaction; F_1,28_ = 12.14, *p* < 0.01) (Fig. 2D) and female (Two-way ANOVA, group x siRNA infusion interaction; F_1,23_ = 12.37, *p* < 0.01) (Fig. 2E) rats, and was attenuated by the EZH2 siRNA infusion into the CeA in both sexes (Fig. 2D, E).

**Fig. 3:**
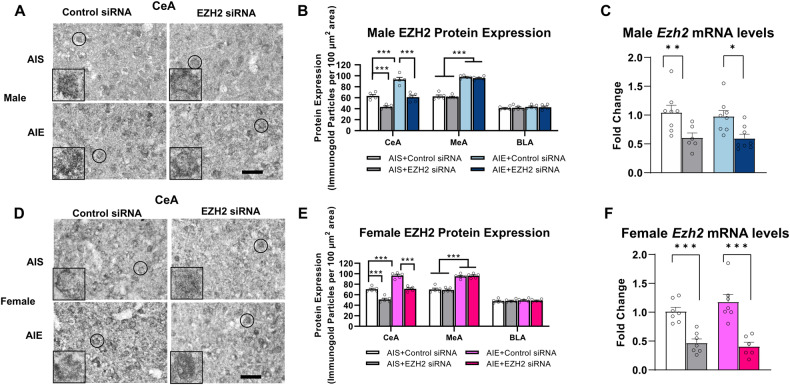
Adolescent intermittent ethanol exposure increases EZH2 in the central (CeA) and medial (MeA) nucleus of amygdala. **A** Representative photomicrograph of gold immunolabeling images in the CeA of various groups in male rats (Scale bar = 40 µm). The inset photograph shows immunogold particles in a single nucleus (marked with circle in each image) at 100x magnification. **B** AIE increases EZH2 protein levels in the CeA and MeA, while knockdown of EZH2 with siRNA (EZH2) infusion into the CeA decreases EZH2 protein levels in the CeA, but not the MeA, in both AIS and AIE adult male rats (*n* = 5/group. **C** qPCR analysis reveals decreased *Ezh2* mRNA expression in the amygdala after EZH2 knockdown in the CeA in male rats (*n* = 6–8/group). **D** Representative photomicrograph of gold immunolabeling images for the CeA in female rats (Scale bar = 40 µm). The inset photograph shows immunogold particles in a single nucleus (marked with circle in each image) at 100x magnification. **E** AIE increases EZH2 expression in the CeA and MeA, while knockdown of EZH2 via siRNA infusion into the CeA decreases EZH2 expression in the CeA, but not the MeA, in both AIS and AIE adult female rats (*n* = 5/group. **F** Similar to male rats, qPCR analysis revealed decreased *Ezh2* mRNA expression in the amygdala after EZH2 knockdown in the CeA in female rats (*n* = 6–7/group). Values are mean ± SEM. Statistical significance was determined with two-way ANOVA followed by Tukey’s post hoc test (*p *<* 0.05, **p *<* 0.01, ***p *<* 0.001). The individual value for each rat is represented by a circle dot on the bar diagram. AIS Adolescent intermittent saline, AIE Adolescent intermittent ethanol.

We validated the above findings of EZH2 protein levels of immunofluorescent staining using a gold immunolabeling procedure. We observed that for male rats, there is an increase in EZH2 protein levels in both the CeA (Two-way ANOVA, effect of adolescent exposure; F_1,16_ = 60.0, *p* < 0.001) and MeA (Two-way ANOVA, effect of adolescent exposure; F_1,16_ = 392.9, *p* < 0.001), but not in the BLA (Fig. 3A, B). EZH2 protein levels were also increased in both the CeA (Two-way ANOVA, effect of adolescent exposure; F_1,16_ = 100.8, *p* < 0.001) and MeA (Two-way ANOVA, effect of adolescent exposure; F_1,16_ = 148.6, *p* < 0.001), but not in the BLA of female rats (Fig. 3D, E). We also found that EZH2 siRNA infusion into the CeA significantly decreased EZH2 protein levels (Males: two-way ANOVA, effect of siRNA infusion; F_1, 16_ = 71.6, *p* < 0.001; Females: two-way ANOVA, effect of siRNA infusion; F_1, 16_ = 93.9, *p* < 0.001) in AIS rats and normalized EZH2 expression in CeA of AIE rats (Fig. 3B, E). EZH2 siRNA infusion also significantly decreased EZH2 mRNA levels in the adult amygdala of AIS and AIE male (Two-way ANOVA, effect of siRNA infusion; F_1, 26_ = 16.9, *p* < 0.001) and female (Two-way ANOVA, effect of siRNA infusion; F_1, 23_ = 53.9, *p* < 0.001) rats (Fig. 3C, F).

### EZH2 knockdown in the CeA prevents decreased *Arc* expression in both sexes

**Fig. 4:**
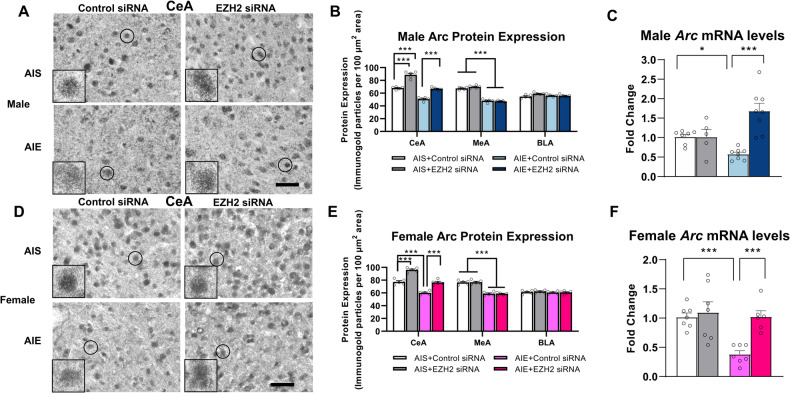
EZH2 knockdown prevents decreases in Arc protein and mRNA levels after AIE in adulthood in both male and female rats. **A** Representative photomicrograph of Arc gold immunolabeling images of the central nucleus of amygdala (CeA) of male rats. Scale bar = 40 µm. Inset photograph shows immunogold particles in a single nucleus (marked with circle in each image) at 100x magnification. **B** Arc protein expression was decreased after AIE in the CeA and MeA. Decrease in Arc protein expression in the CeA was prevented by EZH2 knockdown in the CeA of AIE male rats (*n* = 5/group). **C** *Arc* mRNA levels were decreased in the amygdala after AIE, and this was prevented by EZH2 knockdown in the CeA in male rats (*n* = 5-8/group). **D** Representative photomicrographs of gold immunolabeling images of the CeA of female rats (Scale bar = 40 µm). The inset photograph shows immunogold particles in a single nucleus (marked with circle in each image) at 100x magnification. **E** Arc protein levels were decreased after AIE in the CeA and MeA, and decrease in Arc protein levels in the CeA was prevented by EZH2 knockdown in the CeA of AIE female rats (*n* = 5/group). **F** *Arc* mRNA was decreased in the amygdala after AIE, and this was prevented by EZH2 knockdown in the CeA in female rats (*n* = 6-7/group). Values are mean ± SEM. Statistical significance was determined with two-way ANOVA followed by Tukey’s post hoc test (*p < 0.05, ***p < 0.001). The individual value for each rat is represented by a circle dot on the bar diagram. AIS Adolescent intermittent saline, AIE Adolescent intermittent ethanol.

We evaluated whether knockdown of EZH2 in the CeA in adulthood would restore *Arc* expression. EZH2 siRNA infusion into the CeA prevented decreased *Arc* mRNA expression in both male (Fig. 4C, Two-way ANOVA, interaction; F_1,24_ = 15.4, *p* < 0.001) and female (Fig. 4F, Two-way ANOVA, interaction; F_1,23_ = 5.9, *p* = 0.024) amygdala. We measured Arc protein levels and found that AIE significantly decreased Arc protein levels in both the CeA (Two-way ANOVA, effect of adolescent exposure; F_1,16_ = 174.2, *p* < 0.001) and MeA (Two-way ANOVA, effect of adolescent exposure; F_1,16_ = 498.3, *p* < 0.001) of male rats. EZH2 siRNA infusion into CeA prevented decreased Arc protein levels in male CeA (Two-way ANOVA, effect of siRNA infusion; F_1, 16_ = 153.5, *p* < 0.001) but not the MeA (Fig. 4A, B). Similar findings were observed in females as AIE significantly decreased Arc protein levels in both the CeA (effect of adolescent exposure; F_1, 16_ = 119.3, *p* < 0.001) and MeA (effect of adolescent exposure; F_1, 16_ = 189.1, *p* < 0.001). Infusion with EZH2 siRNA into CeA prevented decreased Arc protein levels in female CeA (Two-way ANOVA, effect of siRNA infusion; F_1, 16_ = 109.7, *p* < 0.001) but not MeA (Fig. 4D, E). Together, these results suggest that AIE caused reductions in both the mRNA and protein levels of Arc, which were normalized by a single infusion of EZH2 siRNA into CeA of both sexes.

### EZH2 regulates Arc expression through epigenetic regulation at *Arc* SARE after AIE in both sexes

**Fig. 5:**
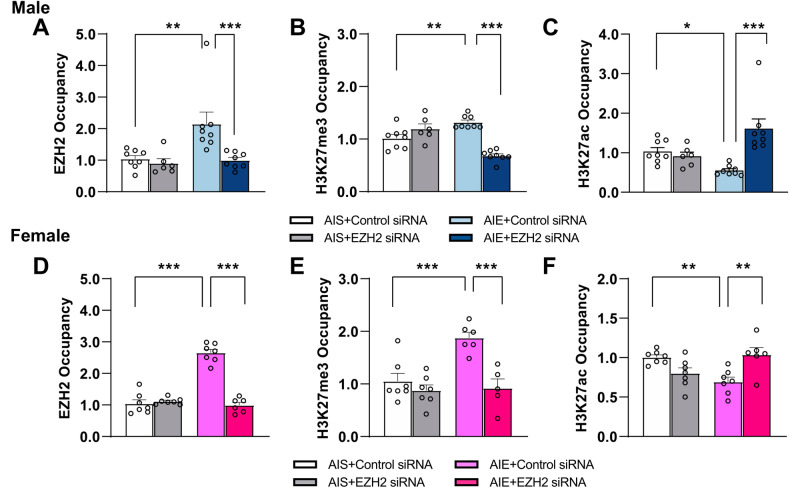
EZH2 knockdown prevents AIE-induced repressive epigenetic remodeling at the *Arc* SARE site in the amygdala of male and female adult rats. AIE increases EZH2 (**A**) and H3K27me3 (**B**) occupancy (fold change) associated with the *Arc* SARE site in adulthood. Knockdown of EZH2 in the central nucleus of amygdala (CeA) prevents these changes in the amygdala of adult male rats (*n* = 6–8/group). **C** AIE decreases H3K27ac associated with the *Arc* SARE, and knockdown of EZH2 in the CeA prevents decreases in H3K27ac in the amygdala of adult male rats (*n* = 6–8/group). AIE increases EZH2 (**D**) and H3K27me3 (**E**) occupancy (fold change) associated with the *Arc* SARE, and knockdown of EZH2 in the CeA prevents these changes in the amygdala of female rats (*n* = 5-7/group). **F** AIE decreases H3K27ac associated with the *Arc* SARE, and knockdown of EZH2 in the CeA prevents decreases in H3K27ac in the amygdala of female rats (*n* = 6–7/group). Values are mean ± SEM. Statistical significance was determined with two-way ANOVA followed by Tukey’s post hoc test (*p < 0.05, **p < 0.01, ***p < 0.001). The individual value for each rat is represented by a circle dot on the bar diagram. AIS Adolescent intermittent saline, AIE Adolescent intermittent ethanol.

We have previously demonstrated that H3K27me3 is increased at the *Arc* SARE via decreased occupancy of lysine demethylase 6B (KDM6B) in the amygdala of AIE male adult rats [26]. Here, we extended these findings by examining the role of histone methyltransferase EZH2 in the regulation of H3K27 methylation and acetylation at the *Arc* SARE that may be responsible for AIE-induced reductions in Arc expression in the amygdala of both adult male and female rats. We found increased occupancy of EZH2 at the *Arc* SARE in the amygdala of both male (Fig. 5A, Two-way ANOVA, interaction; F_1,26_ = 5.1, *p* = 0.032) and female (Fig. 5D, Two-way ANOVA, interaction; F_1,23_ = 76.8, *p* < 0.001) AIE rats, which is consistent with our previous findings in human postmortem amygdala of early age onset of AUD [25] and with global increases in EZH2 protein levels in the amygdala after AIE in adulthood (Fig. 1C, H). We next evaluated H3K27me3, the catalytic product of EZH2, and found increased H3K27me3 occupancy at the *Arc* SARE in the amygdala of both male (Fig. 5B, Two-way ANOVA, interaction; F_1,26_ = 43.5, *p* < 0.001) and female (Fig. 5E, Two-way ANOVA, interaction, F_1,21_ = 8.2, *p* < 0.01) AIE rats. Because H3K27ac is a mark of active enhancers (33), we evaluated H3K27ac status at the *Arc* SARE. We found decreased H3K27ac occupancy at the *Arc* SARE in the amygdala of both male (Fig. 5C, Two-way ANOVA, interaction; F_1,26_ = 14.9, *p* < 0.001) and female (Fig. 5F, Two-way ANOVA, interaction; F_1,23_ = 17.8, *p* < 0.001) AIE rats. Knockdown of EZH2 in the CeA prevented increased EZH2, increased H3K27me3, and decreased H3K27ac occupancy at the *Arc* SARE in the amygdala of both sexes (Fig. 5).

## Discussion

**Fig. 6:**
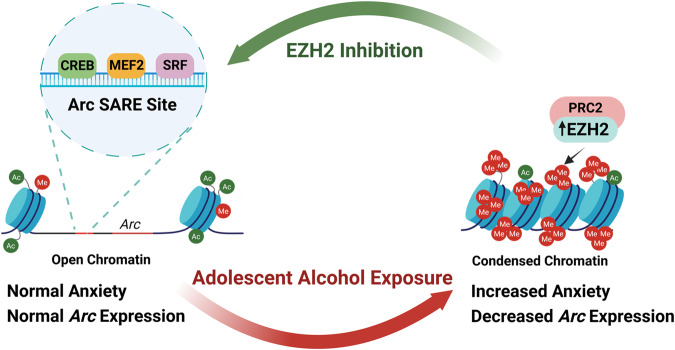
Working model of the role of EZH2 in regulating epigenetic mechanisms and behavioral changes in adulthood after adolescent alcohol exposure. Adolescent alcohol exposure causes an increase in EZH2, which increases H3K27me3 at the *Arc* SARE site, leading to chromatin remodeling that results in decreased Arc mRNA and protein levels in the amygdala of adult male and female rats. Interestingly, these molecular changes induced by EZH2 in the CeA are causally related to AIE-induced anxiety-like behaviors during adulthood in both male and female rats. Interestingly, EZH2 occupancy is also increased at the ARC SARE site in the postmortem amygdala of subjects with early age of onset of alcohol use disorder as compared with control subjects (25). Together, these results suggest that EZH2 can serve as an important epigenetic target for developing drugs to treat or prevent adult psychopathology after adolescent alcohol exposure.

The present study describes a direct role of EZH2 in regulating an anxiety-like phenotype in adulthood that develops after AIE in both male and female rats. This finding confirms the results of our earlier research on early onset AUD human postmortem amygdala [25] that EZH2 regulates *Arc* expression through interactions with the SARE site and demonstrates a unified mechanism for epigenetic dysregulation via EZH2 across both species and sexes (Fig. 6).

### Sex similarities and differences in anxiety-like behavior after adolescent alcohol exposure

Sex differences in the development of AUD and anxiety-like disorders is an area of growing interest. Epidemiological research has demonstrated that men are more likely to have an AUD than women (16.7% and 9.0% respectively), although the rate at which women are being diagnosed with AUD is increasing at a higher rate than that of men (84% vs 35%, respectively) [7, 8]. Adolescents primarily consume alcohol by binge consumption at equal rates between sexes (11.3% for males and 11.4% for females) [37]. While adult women have traditionally lagged behind men in binge drinking, a newer meta-analysis of six national surveys has demonstrated an increasing prevalence of binge drinking among women [38]. Taken together, this research suggests that binge alcohol consumption is similar between men and women during adolescence and that rates of AUD due to adolescent alcohol exposure are beginning to equalize. However, the shared mechanism that increases the risk of developing an AUD following adolescent alcohol consumption has not been fully elucidated. Our current study using a controlled experimental adolescent binge model suggests that adolescent alcohol exposure disrupts normal development processes in the amygdala through a similar epigenetic mechanism in both sexes and can increase the risk of developing an anxiety disorder later in life.

The rodent literature paints a more complex story. One study in C57BL/6 J mice demonstrated that adolescent binge drinking led to higher alcohol consumption in adulthood, but that females were more vulnerable to this effect than males [39]. Another study showed that AIE exposure increased anxiety-like behavior during withdrawal in adolescent male and female mice, but that a stress challenge was more likely to increase adult ethanol consumption only in female mice [40]. Our results demonstrate that the anxiety-like behavior induced by AIE persists until adulthood in both sexes. We have consistently shown that AIE increases alcohol intake in male adult rats [27, 33, 41, 42]. Future studies will investigate whether alcohol intake is driven by an EZH2 mechanism in both male and female rats after AIE in adulthood.

Stressful life events increase both sexes’ likelihoods of developing an AUD, but the odds are higher for females than for males (3.94 vs 2.51, respectively) [43]. While withdrawal-induced anxiety is well documented [4, 12, 44, 45], the role of adolescent alcohol exposure in subsequent development of anxiety in both sexes is less characterized. Our results indicate that AIE induces anxiety-like behavior in both sexes, which is consistent with previous studies in Sprague-Dawley male and female rats [26, 27, 46] and studies of acute withdrawal after AIE in male and female mice [40]. However, others have reported different findings specific to strain or alcohol exposure paradigm using male Long Evans and Wistar rats that showed either decreased or no changes in adult anxiety-like behavior after AIE [47, 48], which implies that development of an anxiety phenotype after AUD is multifactorial and warrants further investigation. Nonetheless, our results suggest that both males and females exhibit a shared adult anxiety phenotype after AIE.

### Sex similarities and differences in molecular and neurobiological substrates after adolescent alcohol exposure

The amygdala has long been known to be involved in stress and alcohol [16, 17]. In humans, men have been shown to have larger amygdala volumes than women [49], although both sexes show similar amygdala activation following negative stimuli [50]. Rodent studies have shown that there are sex differences in CeA neuronal firing in response to stress and alcohol as male neurons show a greater inhibition by acute ethanol, while female neurons are more responsive to the stress hormone corticosterone [51]. Our results indicate that there is a shared synaptic molecular substrate, Arc, that is decreased after AIE, and these results agree with our findings in human postmortem amygdala of early age onset AUD [25]. Arc is an immediate early gene that is involved in synaptic plasticity, long term potentiation, anxiety, learning, memory, and alcohol consumption [12, 26, 33, 52]. Recently, using a CRISPR-Cas9 approach to lower *Arc* expression in the CeA controls cue related alcohol drinking in mice [53], while using epigenetic targeting editing with dCas9-P300 to increase *Arc* expression in the CeA ameliorated anxiety and alcohol consumption in male adult rats after AIE [33]. Another study found that rescuing *Arc* expression in the nucleus accumbens of *Arc* knockout mice normalized anxiety behaviors in the EPM in males but not in females [54]. However, this sex difference did not apply to reductions in novelty discrimination, which occurred in both sexes [54]. This suggests that while *Arc* expression may differ by region and species, there are common responses to environmental and stressful stimuli which are supported by our finding that restoration of *Arc* expression with EZH2 knockdown in the CeA prevents AIE-induced anxiety-like behavior in both male and female adult rats.

### EZH2 as a central mediator of changes in Arc expression induced by adolescent alcohol exposure

Epigenetic changes occur throughout adolescent development and are required for normal brain connectivity and physiology [55]. The PRC2 complex, which includes EZH2, plays an important role in mediating brain development [30]. Further, mutations in EZH2 are associated with Weaver syndrome which disrupts normal development during childhood and adolescence [56]. We previously reported that EZH2 is recruited to the *ARC* SARE site and that this corresponds to increased repressive H3K27me3 and decreased *ARC* expression in early onset human postmortem amygdala in both sexes [25]. Here, we report that this process also occurs in rodents and that decrease in *Arc* expression is linked mechanistically to increased EZH2 and H3K27me3 and decreased H3K27ac associated with the SARE site. Other reports have suggested that redistribution of EZH2 binding across the genome disrupts key genes involved in brain development, leading to phenotypic changes [57]. In a mouse embryonic stem cell model of alcohol exposure, increased EZH2 is associated with increased H3K27me3 at various developmental gene regulatory elements [58]. We also observed that knockdown of EZH2 prevents AIE-induced anxiety-like behavior and reductions in Arc expression in the amygdala of both male and female adult rats. It has been shown that increasing miR-101a-3p expression in the amygdala increases anxiety-like behavior, which is at least partially mediated via changes in EZH2 expression in an inbred strain of rat that has a high-response to novelty [32]. Thus, it is evident that EZH2 plays a critical role in behavior and gene expression that is important for normal development; furthermore, our results suggest that this is disrupted by AIE in both sexes. We recently demonstrated that epigenomic targeting of the *Arc* SARE site with dCas9-P300 and dCas9-KRAB bidirectionally reduces or induces anxiety-like behaviors in AIE and control male rats, respectively [33]. Here, we identified EZH2 as an epigenetic target that regulates histone acetylation and methylation marks at the *Arc* SARE site and controls AIE-induced anxiety-like behaviors in adulthood in both sexes.

### Up regulation of EZH2 expression on PKC-δ positive GABAergic neurons after adolescent alcohol exposure in adulthood

The CeA contains two major nuclei referred to as medial (CeM) and lateral (CeL) CeA which have been shown to interact in emotional processing, such as fear and anxiety [22, 59, 60]. More than 50% cells in the CeL are PKC-δ positive GABAergic cells [60] and inhibitory inputs to CeM appear to be important in the regulation of anxiety phenotype [59]. Activation of PKC-δ positive GABAergic inhibitory neurons using optogenetic manipulation in the CeL produces anxiolytic effects and inhibited consummatory behaviors in animal models [61]. The present study also provides evidence that AIE increased EZH2 protein levels on PKC-δ positive GABAergic neurons in the CeA and MeA of male and female rats, which is consistent with a growing body of literature that these neurons are important in regulating responses to alcohol and anxiety behaviors [22, 62]. More broadly, GABAergic neurons regulate inhibitory function in the CeA and control anxiety phenotypes [21, 22, 61]. Transcriptomic changes in these cells in the CeA are sensitive to ethanol withdrawal [62]. Our data suggest the possibility that higher EZH2 levels may decrease the inhibitory response of PKC-δ positive GABAergic neurons in the CeA and contribute to regulation of anxiety phenotype after AIE in adulthood. However, the current study only provides a basis that EZH2 in PKC- δ GABAergic neurons may be involved in these behaviors, given technical limitations with the experimental manipulation of EZH2 only in PKC-δ GABAergic neurons via siRNA. Future studies are needed to establish the link between EZH2 mediated epigenetic mechanisms and synaptic regulation in PKC-δ positive GABAergic neurons in CeA to AIE-induced anxiety-like behaviors in both sexes.

## Conclusion

This study presents a unified epigenetic mechanism mediated by EZH2 in the CeA that drives anxiety-like behavior after adolescent alcohol exposure in both male and female rats and provides a mechanistic basis (Fig. 6) for our earlier findings in human postmortem amygdala from individuals that developed early onset AUD [25]. This suggests that there are shared neural substrates between sexes and species that are dysregulated by adolescent alcohol exposure, and they change epigenetic regulation of genes associated with synaptic plasticity, such as *Arc*. Further, these data implicate EZH2 as a novel potential target for the development of therapeutics for adult psychopathology after adolescent alcohol consumption.

## Data Availability

The data that supports the findings of this study is provided in the manuscript. The raw data from the corresponding author is available upon reasonable request.
